# Predicting the Intention to Use Generative Artificial Intelligence for Health Information: Comparative Survey Study

**DOI:** 10.2196/75648

**Published:** 2026-01-28

**Authors:** Jörg Matthes, Anne Reinhardt, Selma Hodzic, Jaroslava Kaňková, Alice Binder, Ljubisa Bojic, Helle Terkildsen Maindal, Corina Paraschiv, Knud Ryom

**Affiliations:** 1 Department of Communication, University of Vienna, Waehringer Street 29, Vienna, 1090, Austria, 43 14277493; 2 Department of Media and Communication, Ludwig-Maximilians-Universität München, Munich, Germany; 3 Digital Society Lab, Institute for Philosophy and Social Theory, University of Belgrade, Belgrade, Serbia; 4 Institute for Artificial Intelligence Research and Development of Serbia, Novi Sad, Serbia; 5 Department of Public Health, Aarhus University, Aarhus, Denmark; 6 Laboratoire Interdisciplinaire de Recherche Appliquée en Économie de la Santé (LIRAES), Université Paris Cité, Paris, France

**Keywords:** generative AI, artificial intelligence, health information–seeking, UTAUT2, Unified Theory of Acceptance and Use of Technology 2, AI adoption

## Abstract

**Background:**

The rise of generative artificial intelligence (AI) tools such as ChatGPT is rapidly transforming how people access information online. In the health context, generative AI is seen as a potentially disruptive information source due to its low entry barriers, conversational style, and ability to tailor content to users’ needs. However, little is known about whether and how individuals use generative AI for health purposes, and which groups may benefit or be left behind, raising important questions of digital health equity.

**Objective:**

This study aimed to assess the current relevance of generative AI as a health information source and to identify key factors predicting individuals’ intention to use it. We applied the Unified Theory of Acceptance and Use of Technology 2, focusing on 6 core predictors: performance expectancy, effort expectancy, facilitating conditions, social influence, habit, and hedonic motivation. In addition, we extended the model by including health literacy and health status. A cross-national design enabled comparison across 4 European countries.

**Methods:**

A representative online survey was conducted in September 2024 with 1990 participants aged 16 to 74 years from Austria (n=502), Denmark (n=507), France (n=498), and Serbia (n=483). Structural equation modeling with metric measurement invariance was used to test associations across countries.

**Results:**

Usage of generative AI for health information was still limited: only 39.5% of respondents reported having used it at least rarely. Generative AI ranked last among all measured health information sources (mean 2.08, SD 1.66); instead, medical experts (mean 4.77, SD 1.70) and online search engines (mean 4.57, SD 1.88) are still the most frequently used health information sources. Despite this, performance expectancy (b range=0.44-0.53; all *P*<.001), habit (b range=0.28-0.32; all *P*<.001), and hedonic motivation (b range=0.22-0.45; all *P*<.001) consistently predicted behavioral intention in all countries. Facilitating conditions also showed small but significant effects (b range=0.12-0.24; all *P*<.01). In contrast, effort expectancy, social influence, health literacy, and health status were unrelated to intention in all countries, with one marginal exception (France: health status, b=−0.09; *P*=.007). Model fit was good (comparative fit index=0.95; root mean square error of approximation=0.03), and metric invariance was confirmed.

**Conclusions:**

Generative AI use for health information is currently driven by early adopters—those who find it useful, easy to integrate, enjoyable, and have the necessary skills and infrastructure to do so. Cross-national consistency suggests a shared adoption pattern across Europe. To promote equitable adoption, communication efforts should focus on usefulness, convenience, and enjoyment, while ensuring digital access and safeguards for vulnerable users.

## Introduction

### Background

When people look for health information today, they no longer only consult physicians, pharmacists, or search engines. Increasingly, they also encounter generative artificial intelligence (AI) tools such as ChatGPT or the World Health Organization (WHO)’s chatbot Sarah, which simulate human-like conversations and provide instant responses. These tools promise a new way of accessing medical knowledge: fast, convenient, and interactive. At first glance, this accessibility seems to hold great potential for reducing barriers to health information, therefore directly impacting digital health equity—defined as equitable access to and use of digital health information technology that supports informed decision-making and enhances health [1].

However, the picture is more complex. On the one hand, generative AI can offer cost-free entry points (eg, basic versions of ChatGPT or automatically displayed answers in Google search via Google’s Gemini), deliver content in multiple languages, and rephrase complex medical concepts into more understandable terms. In doing so, it could strengthen patient education, address health inequalities, and help bridge communication gaps between citizens and health care providers [2,3]. On the other hand, effective use still depends on internet-enabled devices and adequate digital skills, which are not equally distributed. As a result, the very technology that appears open and inclusive may also risk exacerbating existing digital divides [4].

Moreover, unlike other types of information, health-related questions are often sensitive and personal. At the same time, the inner workings of generative AI remain opaque, and the accuracy of its outputs is not guaranteed [5]. All these tensions raise important questions about adoption: Who is most likely to turn to generative AI for health information, and what factors shape this intention? Moreover, since health communication practices and digital infrastructures differ across countries, cross-national research is urgently needed.

To address these questions, this study draws on the Unified Theory of Acceptance and Use of Technology 2 (UTAUT2) [6]. The model proposes that performance expectancy, effort expectancy, facilitating conditions, social influence, habit, and hedonic motivation shape technology use. We extend this framework by also examining the roles of health literacy and health status in predicting intention to use generative AI for health information. Using cross-national survey data from Austria, Denmark, France, and Serbia, we investigate the drivers of adoption to shed light on both individual and contextual factors that may guide the diffusion of generative AI in health contexts.

### Generative AI as Novel Health Information Source

Generative AI constitutes a potentially disruptive force in the health information ecosystem [7]. However, despite its rapid advancement and widespread availability, empirical research on its role in health information–seeking remains limited [2]. At the same time, broader trends highlight the ongoing digitalization of health-related knowledge acquisition. A representative survey conducted in Germany in 2019 revealed that only 48% of respondents consulted a medical professional for their most recent health issue, while 1 in 3 turned first to the internet [8]. Similar findings show that online sources—particularly search engines—are the primary means of accessing health information, both for caregivers and the general population [9,10]. Family and friends and traditional mass media (eg, print media and health-related TV programming) rank behind medical professionals and online sources [8].

The introduction of generative AI tools like ChatGPT may shift these established hierarchies. Unlike static web content or conventional search engines, generative AI enables dialogic, personalized interactions that simulate human conversation. These features may position generative AI as a compelling alternative to established online and offline health information sources. However, current evidence suggests that trust in generative AI—especially regarding complex health-related issues—is still limited [11], which might restrict its present adoption potential to early adopters [2]. This raises questions about how generative AI integrates into the broader ecosystem of health information sources.

To address this gap, we first explore: How does the use of generative AI for health information–seeking compare to that of more established health information sources?

### Explaining Predictors of Technology Adoption: UTAUT2

The UTAUT2 [6] is one of the most popular models to explain technology adoption. It builds on the technology acceptance model [12], emphasizing perceived usefulness and ease of use, and the initial UTAUT model [13], which added performance expectancy, effort expectancy, facilitating conditions, and social influence as predictors of adoption behavior. UTAUT2 extends these frameworks to consumer contexts by incorporating hedonic motivation and habit [6,14] . The UTAUT2 model has demonstrated its versatility in explaining the adoption of diverse eHealth technologies, such as wearable devices [15], health websites [16], and health apps [17]. Additionally, recent studies have highlighted its relevance in understanding the uptake of generative AI technologies [18-21], showcasing its capacity to extend beyond traditional eHealth domains. However, so far, studies on predictors of usage intentions in the context of AI health information–seeking are lacking.

Performance expectancy, a central construct in the UTAUT2 framework, reflects the belief that using technology will lead to performance benefits [22]. In the context of health information–seeking using generative AI, performance expectancy is shaped by users’ perceptions of how effectively these tools can enhance their own life, including aspects such as health–decision-making and task efficiency [23]. Consequently, as users anticipate greater usefulness from adopting generative AI as a health information source, their intention to use such technologies strengthens [18-20,24]. Based on this, we propose the following hypothesis: “the higher the performance expectancy, the stronger the intention to use generative AI for health information–seeking” (H1)*.* Effort expectancy, closely tied to ease of use, emphasizes simplicity in technology adoption [13]. Generative AI tools like ChatGPT benefit from high effort expectancy when users find them intuitive and easy to integrate into their workflows, particularly during the early adoption phase [18,19,23,24]. Addressing usability concerns early can reduce resistance and build user confidence, strengthening behavioral intention [23,25]. Therefore, we propose that “the higher the effort expectancy, the stronger the intention to use generative AI for health information–seeking” (H2). Facilitating conditions refer to the resources, skills, and support necessary for using technology [22]. These include training, knowledge, technical assistance, and system compatibility, which significantly enhance behavioral intention and usage [6,25]. In technologically mature settings, facilitating conditions are critical for sustained adoption and user satisfaction [26]. In line with the UTAUT2, we hypothesize that “the better the facilitating conditions, the stronger the intention to use generative AI for health information–seeking” (H3). Social influence indicates the perception that peers, such as family, friends, or colleagues, believe one should adopt a technology [22]. It plays a crucial role in early adoption, where external validation often outweighs personal experience [6]. Positive reinforcement within social or professional networks can normalize usage [18,24,25,27]: If people perceive that their peers already use generative AI for health information–seeking, their own intention to do so might increase as well. We, therefore, propose that “the greater the perceived social influence, the stronger the intention to use generative AI for health information–seeking” (H4)*.* Habit specifies the extent to which behavior becomes automatic through repetition and prior use [26]. It strongly influences behavioral intention and long-term adoption, emphasizing the importance of regular engagement with technology [6,28]. For generative AI as a health information source, fostering habitual use can solidify its integration into daily routines and enhance sustained adoption [25]. This leads us to state, “the more it is a habit to use generative AI, the stronger the intention to use generative AI for health information–seeking” (H5). Hedonic motivation refers to the enjoyment or pleasure derived from using technology, particularly relevant in consumer contexts [26]. It directly impacts behavioral intention, especially for technologies involving entertainment or leisure [29]. For generative AI like ChatGPT, it can be expected that the interaction is perceived as fun or entertaining, which can boost user engagement and drive adoption [30]. Accordingly, we suggest the following hypothesis: “the higher the hedonic motivation, the stronger the intention to use generative AI for health information–seeking” (H6)*.*

### Influence of Health Literacy and Health Status

With the growing integration of digital tools into everyday lives, the role of health literacy in online health information–seeking has garnered increasing attention. Health literacy has been conceptualized as an individual’s capacity to search, access, comprehend, and critically evaluate health information, as well as to use the acquired knowledge to effectively address health-related issues [31,32]. Digital health literacy refers to these abilities in the context of digital environments [33-35]. Generally, low health literacy scores have been associated with undesirable health outcomes [36].

Research suggests that low levels of health literacy are associated with decreased trust in online health resources [37], including the outputs of AI tools [38], and lower overall adoption of online health technologies [39]. Furthermore, initial studies indicate a positive association between health literacy levels and attitudes toward the use of AI tools for medical consultations [40].

On the other hand, individuals with higher levels of health literacy are generally better equipped to critically evaluate online health information and scrutinize it in greater detail [37,41]. This heightened evaluative capacity could make them more aware of the limitations and potential risks of generative AI outputs, such as inaccurate information, bias, data privacy concerns, or oversimplified medical advice [42]. Moreover, individuals with higher health literacy are more likely to trust and use high-quality medical online resources, whereas those with limited health literacy prefer accessible but potentially less reliable sources [43]. In this context, outputs from generative AI might be perceived as lower-quality sources by highly digital health–literate individuals. As a result, while higher health literacy could foster openness to using generative AI for health purposes, it might also lead to greater skepticism or hesitancy in relying on these tools. Nonetheless, there is not enough research in the context of generative AI specifically to make conclusive predictions.

Another well-established factor in online health information–seeking, yet underexplored in the context of AI, is individuals’ health status: Studies suggest that people with poor health are significantly more likely to consult the internet for health information compared to those with good health [44,45]. Being chronically ill has also been associated with increased reliance on internet-based technologies for health-related purposes [46]. This relationship can be explained by the fact that individuals in poor health often experience greater health-related concerns, which in turn heightens their motivation to seek information online.

Given these complex relationships, we propose the second research question: How does health literacy and health status influence the intention to seek health information using generative AI?

### Cross-National Comparison

In this study, we investigate the predictors of generative AI adoption for health information–seeking across 4 European countries: Austria, Denmark, France, and Serbia. While these countries share certain similarities, they also display notable differences that could shape the strength of the UTAUT2 predictors on the intention to use generative AI for health purposes. Thus, this cross-national approach ensures that the observed effects are generalizable and not confined to specific national contexts or unique country conditions.

The selected countries share two key characteristics. First, all 4 countries provide universal health coverage, ensuring broad access to health care services for their populations. Second, a significant portion of health care expenditure in these countries is publicly funded [47-50].

Despite these commonalities, there are also critical factors that differ among the countries and may shape the predictors of generative AI adoption. On the one hand, variations in digital infrastructure could significantly impact facilitating conditions, effort expectancy, and social influence as predictors of generative AI use. Denmark consistently ranks among Europe’s most digitally advanced nations, boasting high internet penetration and widespread adoption of e-health solutions [51]. This strong digital ecosystem likely enhances the perceived ease of use and social endorsement of generative AI. In contrast, Austria, France, and Serbia exhibit more moderate levels of digital adoption in the context of health information, which may limit the perceived use and social norms regarding such technologies [51].

On the other hand, access to and trust in health care providers vary significantly across these countries, potentially influencing performance expectancy and social influence. In nations with robust health care systems—characterized by a high availability of medical professionals and easy access to care—individuals are more likely to rely on doctors for health advice, as they are often viewed as the most trusted source of health information [8]. Denmark exemplifies this with its high levels of public trust in the health care system [52], which may reduce the perceived benefits and social norms around using generative AI for health purposes. Conversely, in western Balkan countries like Serbia, studies report generally low levels of trust in the health care system [53]. In such contexts, individuals may be more inclined to seek alternative information sources, potentially amplifying the perceived benefits of generative AI use.

By examining these diverse national contexts, this study not only tests the universality of the UTAUT2 model but also deepens our understanding of the contextual factors that shape generative AI adoption for health purposes. We ask: How do the predictors of generative AI use for health information–seeking differ across Austria, Denmark, France, and Serbia?

## Methods

### Ethical Considerations

Before data collection, the study received ethical approval from the institutional review board of the Department of Communication at the University of Vienna (approval ID: 1205). All participants provided written informed consent prior to participating in the study. The data were collected in anonymized form and no personal identifiers were recorded or stored. Participants received a compensation of €1.50 (US $1.74) for completing the study through the panel provider.

### Recruitment

Recruitment of participants occurred during September 2024 via *Bilendi*, an international panel provider. *Bilendi* recruited the participants via email. The panel is checked for quality and attendance on a regular basis. The study was conducted in Austria, France, Denmark, and Serbia, with participants randomly selected to achieve samples representative of age, gender, and educational background. The provider’s panel sizes in the respective countries were as follows: Austria: n=60,000; Denmark: n=90,000; France: n=815,000; and Serbia: n=15,100. Per country, the study aimed to reach 500 participants.

Inclusion criteria required participants to be aged between 16 and 74 years. Additionally, respondents who completed the survey in less than one-third of the median completion time (speeders) were excluded. Completion rates (excluding screened-out participants) were high across all 4 countries, ranging from 84.3% to 89.8%. Further details on survey design, administration, and response rates are provided in the CHERRIES (Checklist for Reporting Results of Internet E-Surveys) checklist (Checklist 1).

### Procedure and Measures

#### Overview

The study consisted of two components. The first component, a survey, investigated predictors of the intention to use generative AI for health information–seeking. The second component, an experimental study, explored the influence of disease-related factors on these intentions [54]. To ensure respondents shared a common understanding of the concept, the survey began with a short definition of “generative AI,” describing it as technologies that engage in natural language conversations with users and generate responses in real-time. Examples such as ChatGPT, Google’s Gemini, and Microsoft Copilot were provided.

The original questionnaire was developed in English and subsequently translated into German, French, Danish, and Serbian. Each translation was performed by a bilingual team member and back-translated into English by a different native speaker to ensure conceptual equivalence with the original items.

This study focused on the following constructs (a complete item list with descriptive analysis and construct reliability values can be found in Multimedia Appendix 1).

#### Dependent Variables

##### Sources of Health Information–Seeking

Participants rated the frequency with which they use 8 health information [55] sources on a 7-point Likert scale (1 = “never” to 7 = “very often”). The sources included conversations with medical professionals, pharmacists, and family or friends, as well as books, mass media, internet search engines (eg, Google and Ecosia), and generative AI.

##### AI Usage Intentions

Participants’ willingness to use generative AI [25] for health information–seeking was assessed using 3 items (eg, “I intend to use generative AI for health information seeking”), rated on a 7-point Likert scale (1 = “strongly disagree” to 7 = “strongly agree”).

### UTAUT2 Predictor Variables

All UTAUT2 model [6,25,56,57] predictors were measured on a 7-point Likert scale (1 = “strongly disagree” to 7 = “strongly agree”). The predictors have been described below.

#### Performance Expectancy

Perceived benefits of using generative AI for health information–seeking were measured with 4 items (eg, “Using generative AI would save me time when researching health topics”).

#### Effort Expectancy

The perceived ease of using generative AI as a health information source was assessed with 4 items (eg, “Learning to use generative AI for health information–seeking seems easy for me”).

#### Social Influence

Three items measured the extent to which participants felt that important others encouraged their use of generative AI for health information–seeking (eg, “People who are important to me think that I should use generative AI for health-information seeking”).

#### Hedonic Motivation

The enjoyment of using generative AI for health information–seeking was assessed with 3 items (eg, “I think using generative AI for health-information seeking could be fun”).

#### Facilitating Conditions

Participants’ perceptions of available resources and support for using generative AI to seek health information were measured with 4 items (eg, access to devices and reliable internet, and knowledge).

#### Habit

The extent to which turning to generative AI when seeking health information had become a habitual behavior was measured with 3 items (eg, “I automatically turn to generative AI whenever I have questions about my health”).

### Model Extension Variables

#### Health Literacy

Health literacy [58] was assessed with 10 items, asking participants to rate their confidence in tasks such as finding understandable health information. Responses were recorded on a 4-point Likert scale (1 = “not at all true” to 4 = “absolutely true”).

#### Health Status

We measured participants’ health status using 1 item (“How would you describe your current health status?”; 1 = “very poor” to 7 = “very good”).

### Control Variables: Sociodemographic Variables

We further measured participants’ age, gender, and educational level.

### Statistical Analysis

#### Power

An a priori power analysis was conducted to determine the required sample size for structural equation modeling. Assuming an anticipated effect size of 0.25, a desired statistical power of 0.95, and a significance level of .05, the analysis indicated that a minimum of 391 participants per country would be necessary to detect the hypothesized effects [59].

#### Analytical Plan

We used AMOS version 26 (IBM Corp) to run a latent variable, multigroup structural equation model using a maximum-likelihood estimator with full information. We computed the comparative fit index, the Tucker-Lewis Index, the chi-square to degrees of freedom ratio (χ^2^/df), and the root mean square error of approximation. We also secured metric measurement invariance to be able to compare the paths across countries. We controlled for age, gender, and education (binary coded).

## Results

### User Statistics

In total, data were collected from 1990 respondents, comprising 502 from Austria, 507 from Denmark, 498 from France, and 483 from Serbia. The overall mean age of participants was 45.1 (SD 15.7) years, with 50.2% (n=998) identifying as female participants. In terms of educational attainment, 83.8% (n=1634) of the sample reported completing at least a medium or higher level of education (secondary level II or higher). Furthermore, 87.4% (n=787) of respondents indicated prior use of generative AI for health information–seeking (at least rarely). Detailed demographic and background characteristics of the sample are summarized in Table 1.

**Table 1.** Descriptive characteristics of survey respondents from Austria, Denmark, France, and Serbia (N=1990; September 2024).

Demographic characteristics	Overall, n (%)	Austria, n (%)	Denmark, n (%)	France, n (%)	Serbia, n (%)
Education^a^					
Secondary I or lower	356 (18.24)	93 (18.6)	136 (26.9)	109 (21.9)	18 (3.7)
Secondary II	1080 (55.36)	303 (60.3)	179 (35.3)	224 (45.0)	374 (77.4)
Tertiary	554 (28.39)	106 (21.1)	192 (37.9)	165 (33.1)	91 (18.8)
Gender					
Female	998 (50.15)	252 (49.8)	251 (49.5)	256 (51.4)	239 (49.5)
Male	992 (49.85)	250 (50.2)	256 (50.5)	242 (48.6)	244 (50.5)
Prior experience^b^					
No	1203 (60.45)	316 (62.9)	328 (64.7)	326 (65.5)	233 (48.2)
Yes	787 (39.54)	186 (37.1)	179 (35.3)	172 (34.5)	250 (51.8)

a Educational attainment was categorized as low (secondary level I or below) and medium or high (secondary level II or higher). In Serbia, however, representativeness was achieved by grouping educational levels into low or medium (secondary level II or below) and high (tertiary education) due to sampling limitations.

b Prior experience: no = “I have never used Generative AI for health information seeking” and yes = “I have used Generative AI for health information seeking at least rarely.”

Statistical tests revealed no significant differences in gender distribution across countries (*χ*²_3_=0.48; *P*=.92) and no significant differences in age (Kruskal-Wallis *χ*²_3_=2.15; *P*=.54). In contrast, educational attainment varied significantly between countries (*χ*²_3_=550.76; *P*<.001), reflecting sampling-related imbalances in Serbia where low versus medium or high education was assessed differently than in the other countries. Finally, prior experience with health information–seeking showed significant country differences (Kruskal-Wallis *χ*²_3_=30.95; *P*<.001), with higher levels reported in Serbia.

### Evaluation Outcomes

#### Descriptive Analysis

**Figure 1.**
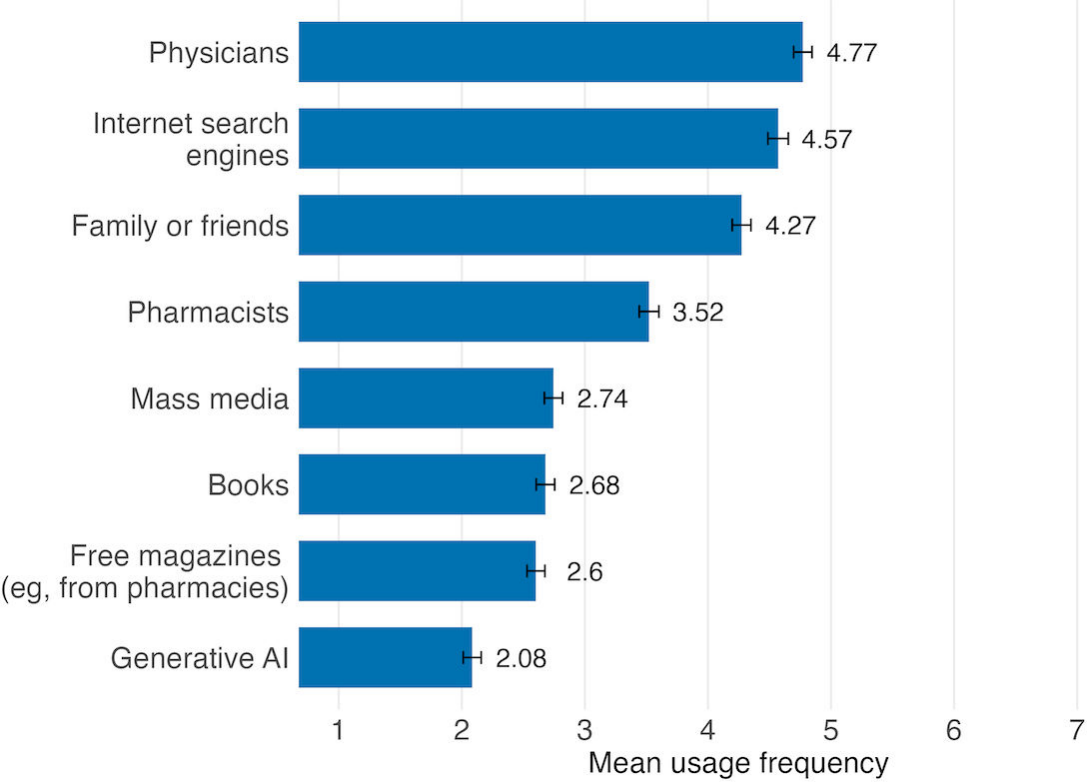
Mean usage frequency of different health information sources among survey respondents (N=1990) in Austria, Denmark, France, and Serbia (95% CI; September 2024). AI: artificial intelligence.

In our first research question, we explored how generative AI compares to more established health information sources in terms of usage frequency across countries. As illustrated in Figure 1, generative AI ranks last among all measured sources, indicating that, as of autumn 2024, it is rarely used for health information–seeking (mean 2.08, SD 1.66). In stark contrast, online search engines like Google are highly used, ranking second with a mean usage frequency of 4.57 (SD 1.88), following conversations with physicians, which hold the top position (4.77, SD 1.70). Family and friends also play a significant role, ranking third (4.27, SD 1.73), alongside pharmacists (3.52, SD 1.81). In comparison, traditional mass media such as TV, newspapers, and magazines are used less frequently (2.74, SD 1.68), as are books (2.68, SD 1.70) and free magazines provided by pharmacies or health insurance companies (2.60, SD 1.65). The relative ranking of information sources was consistent across all 4 countries, with physicians, internet search engines, and family or friends occupying the top positions and generative AI ranking last. However, some variation in mean usage frequencies was observed between countries; detailed country-level results are presented in Multimedia Appendix 2.

#### Model Evaluation

For the hypothesis tests, the results are shown in Table 2. Model fit was good (comparative fit index=0.95; Tucker-Lewis-Index=.93; *χ*^2^/df =2.47, *P*<.001; root mean square error of approximation=0.03, 95% CI 0.03-0.03). We examined the metric measurement invariance of all latent variables by constraining all factor loadings as equal for all 4 countries. When comparing the constrained model to the unconstrained model, we found no significant difference in model fit (*P*=.16). Thus, metric invariance across countries was established.

**Table 2.** Structural equation model predicting the intention to use generative artificial intelligence (AI) for health information among survey respondents in Austria, Denmark, France, and Serbia (N=1990; September 2024).

Predictor variables	Austria^a^			Denmark^b^			France^c^			Serbia^d^		
	b	SE	*P* value	b	SE	*P* value	b	SE	*P* value	b	SE	*P* value
Performance expectancy	0.47	0.05	<.001	0.52	0.05	<.001	.053	0.05	<.001	0.44	0.05	<.001
Effort expectancy	−0.07	0.05	.20	0.03	0.05	.54	−0.11	0.05	.04	-0.02	0.06	.77
Facilitating conditions	0.12	0.04	.01	0.17	0.05	<.001	0.22	0.05	<.001	0.24	0.05	<.001
Social influence	−0.05	0.04	.29	−0.08	0.05	.17	−0.09	0.05	.10	−0.05	0.04	.27
Habit	0.29	0.04	<.001	0.32	0.04	<.001	0.28	0.05	<.001	0.28	0.04	<.001
Hedonic motivation	0.45^e^	0.06	<.001	0.22^f^	0.05	<.001	0.33	0.05	<.001	0.23^f^	0.05	<.001
Health literacy	−0.004	0.09	.97	0.04	0.10	.67	−0.02	0.08	.08	0.08	0.10	.40
Health status	−0.002	0.03	.95	0.02	0.03	.61	−0.09	0.03	.01	−0.05	0.03	.13

a Explained variance=0.84.

b Explained variance=0.80.

c Explained variance=0.86.

d Explained variance=0.79.

e The different subscripts in each row indicate a significant difference between paths (*P*<.05)

f The different subscripts in each row indicate a significant difference between paths (*P*<.05)

In line with H1, we found a highly significant positive association between performance expectancy and the intention to use generative AI for health information–seeking across all 4 countries (Austria: b=0.47, *P*<.001; Denmark: b=0.52, *P*<.001; France: b=0.53, *P*<.001; Serbia: b=0.44, *P*<.001). In contrast, H2 was not supported: effort expectancy showed no significant association with behavioral intention in any of the countries. Turning to H3, results revealed a positive association between facilitating conditions and the intention to use generative AI as a health information source, consistently observed across all 4 contexts (Austria: b=0.12, *P*=.005; Denmark: b=0.17, *P*<.001; France: b=0.22, *P*<.001; Serbia: b=0.24, *P*<.001). By contrast, no support was found for H4: perceived social influence was unrelated to behavioral intention in any of the countries. As predicted in H5, habit was positively associated with behavioral intention to use generative AI for health information–seeking throughout the sample (Austria: *b*=0.29, *P*<.001; Denmark: b=0.32, *P*<.001; France: b=0.28, *P*<.001; Serbia: *b*=0.28, *P*<.001). A similar pattern emerged for H6: hedonic motivation was significantly positively related to behavioral intention in all countries (Austria: b=0.45, *P*<.001; Denmark: b=0.22, *P*<.001; France: b=0.33, *P*<.001; Serbia: b=0.23, *P*<.001).

Finally, with regard to our second research question—which examined whether health literacy and health status predict the intention to seek health information using generative AI—we found no substantial associations. Only in France did health status show a marginal negative effect (b=−0.09; *P*=.007).

## Discussion

### Principal Results

This study investigated the predictors of intention to use generative AI for health information–seeking, drawing on the UTAUT2 framework and expanding it with health literacy and health status. Using cross-national survey data from Austria, Denmark, France, and Serbia, our findings show that generative AI is still only rarely used for health information–seeking. At the same time, performance expectancy, facilitating conditions, habit, and hedonic motivation consistently emerged as significant predictors of behavioral intention, whereas effort expectancy, social influence, health literacy, and health status were not related to intention. These patterns were consistent across all 4 countries, suggesting a robust set of psychological drivers underlying the early adoption of generative AI in health contexts. A detailed examination of these findings is provided as follows.

First, with regard to overall usage patterns, the data shows that generative AI currently plays only a minor role in health information–seeking: 60% of the respondents reported never having used a generative AI tool for health-related questions. This result lends itself to 2 contrasting interpretations.

On the one hand, it challenges the popular narrative that generative AI is rapidly transforming health information–seeking behavior. Instead, the findings align with previous studies, showing that generative AI is currently infrequently used in the context of health information [2]. Traditional sources—such as medical professionals and search engines—continue to dominate [8], underscoring that generative AI has yet to achieve mainstream adoption.

On the other hand, despite persistent concerns about data privacy, algorithmic bias, and accuracy, it is noteworthy that 40% of the respondents have already experimented with generative AI for health purposes. Given that this technology only became widely accessible relatively recently, such early uptake is remarkable. From the perspective of technology adoption models, such as the Rogers Diffusion of Innovations [60], this pattern is characteristic of early adopters. It is therefore plausible to assume that the use of generative AI for health information–seeking will increase further as the technology matures and moves toward mainstream adoption.

To better understand the drivers of future uptake, we applied an extended version of the UTAUT2 model. Our findings confirmed the predictive power of performance expectancy, facilitating conditions, habit, and hedonic motivation. This aligns with prior research on digital health tools, indicating that users value usefulness, access, familiarity, and enjoyment [18,20,24].

In detail, the results show that performance expectancy—the perceived usefulness of the technology—had a strong positive effect on behavioral intention in all four countries. This finding suggests that the more respondents believe generative AI is useful to manage health-related questions, the more they will use it. Thus, if public health stakeholders or developers aim to encourage responsible AI use, they should emphasize the tangible benefits of generative AI, such as 24/7 availability, rapid response times, and the potential for personalized information. Perceived usefulness may also be fostered when individuals try out generative AI for the first time, that is, they learn that they can benefit from the technology.

At the same time, our study challenges established UTAUT2 assumptions. Effort expectancy, often seen as central to technology adoption, was not a relevant factor—possibly due to the intuitive nature of generative AI tools and the ubiquity of basic digital skills [61]. Using generative AI does not require any specific background knowledge beyond opening a webpage. Since online search engines are already the most frequently used health source, the basic skills needed for generative AI are widely present, potentially rendering effort expectancy less decisive.

Taken together, this emerging pattern—the strong effect of performance expectancy and the null effect of effort expectancy—underscores the distinction between usefulness and usability, which are closely related but not identical [62]. Usability refers to the ease of interacting with a system (eg, ease of learning and error prevention), whereas usefulness (utility) captures whether the system provides the functions and information that users actually need. Our findings suggest that in health contexts, utility is the decisive factor: people intend to use generative AI if its outputs are perceived as useful, while usability-related aspects appear less influential.

Importantly, this does not mean that barriers to adoption are absent. Rather, our findings show that they lie not in usability but in facilitating conditions—the structural and contextual resources that enable technology use. Across all countries, the availability of digital infrastructure, devices, and basic knowledge significantly shaped behavioral intention. In other words, while generative AI may be easy to use once accessed, unequal access to the necessary resources continues to pose a substantial adoption barrier. Consequently, facilitating conditions emerge as a key digital health equity concern [4]. Without adequate access, disadvantaged populations may be excluded from benefiting from generative AI, meaning that the technology risks widening rather than narrowing the digital divide in health information–seeking.

We also found that social influence—an important predictor in other studies on AI uptake [18,24]—did not play a meaningful role in shaping behavioral intention. This suggests that health-related information search is a rather personal topic, and that individuals may not always be willing to disclose what kind of information they are looking for. As a result, the intention to use generative AI for health information–seeking is largely independent of peer opinions or social norms.

In contrast, habit consistently predicted behavioral intention across all countries. From this finding, we may conclude that generative AI use for health information is likely to occur automatically, similar to how people use search engines. When individuals feel familiar with a technology, they are more likely to rely on it without conscious deliberation. However, this finding should be interpreted with caution, as the majority of participants had never used generative AI for health purposes. Much of the variance in habit may therefore reflect mere use versus nonuse. Accordingly, variables capturing initial adoption should be clearly distinguished from those measuring habit.

By including health literacy and health status as additional predictors, our study adds a novel dimension to existing research. In contrast to studies showing direct paths between these constructs and online health information–seeking [37,40,46], we found no such association for AI health information–seeking. However, each of these findings carries different implications. First, the absence of a significant association between health literacy and intention indicates that individuals’ ability to understand and evaluate health information was not related to whether they reported turning to generative AI. This finding may suggest that the use of such tools is driven less by informed decision-making and more by general curiosity or interest in new technologies. Importantly, this raises concerns: people with lower health literacy may be just as likely to consult generative AI as those with higher health literacy—despite being less equipped to critically assess its outputs. Given the known risk of AI hallucinations—fabricated or inaccurate information presented in a confident tone [63]—this could lead to misinformation and, in the worst case, harmful health decisions, as users with limited health literacy might find it difficult to distinguish between reliable and misleading content.

Second, the lack of an association between self-reported health status and intention suggests that the current use of generative AI is not primarily driven by medical need or urgency. People do not seem more likely to consult generative AI when facing a health problem; rather, usage may occur proactively or even recreationally. This challenges assumptions that such tools are primarily used in response to a health issue, and it underscores the importance of understanding user motivations beyond immediate health concerns.

Importantly, these patterns were largely consistent across all 4 countries, as confirmed by the measurement-invariant structural model. This cross-national consistency suggests that the psychological drivers of generative AI adoption in health contexts may transcend national boundaries and cultural differences, pointing to a universal set of adoption mechanisms.

### Limitations

Several limitations should be acknowledged. First, due to the cross-sectional nature of this study, no causal conclusions can be drawn. Future research should therefore aim to replicate these findings using experimental or longitudinal designs. Second, we relied on self-reported data, which may be subject to social desirability bias. The use of behavioral data is thus warranted to validate these findings. Third, including additional predictors—such as individual differences or specific concerns—could provide deeper insights into the use of generative AI. Fourth, our comparative findings are based on data from only 4 countries, which limits the ability to conduct multilevel analyses. Also, as in all cross-sectional research, there is a risk of unmeasured third variables. In particular, we did not include AI trust and perceived AI risk. However, these constructs are conceptually close to performance expectancy, as trust reduces uncertainty about the system’s outputs and thereby enhances expected performance gains, whereas perceived risk erodes expected utility. In this sense, they are likely to be partially reflected in the performance expectancy construct already included in our model. That said, and highlighting that our model explains around 80% of the variance, trust and perceived risk could still suppress some of the predictors we have modeled. Thus, future research should include additional constructs outside the UTAUT2 framework [64]. Finally, health status was measured with a single self-rated item. While single-item measures of subjective health may not capture the full complexity of an individual’s medical condition, this approach is widely used in demographic and population health research. Prior work has demonstrated that the self-rated health item is a valid and reliable indicator, predicting key outcomes such as mortality, use of health services, and health expenditures in large-scale surveys [65]. Nevertheless, we acknowledge that a more fine-grained measure (eg, including specific chronic conditions or severity indices) could have provided additional insights, and future studies may benefit from applying such extended health measures.

### Conclusions

This study applied the UTAUT2 model to investigate the factors that drive the use of generative AI for health information–seeking. Although overall usage remains limited, our findings show that performance expectancy, facilitating conditions, habit, and hedonic motivation are positively associated with behavioral intentions. These patterns, observed across all 4 countries—Austria, Denmark, France, and Serbia—suggest that current users of generative AI are likely to be early adopters: individuals who are tech-savvy, curious, and open to innovation. This aligns with the Rogers Diffusion of Innovations theory, which conceptualizes adoption as a gradual process beginning with a small, innovation-oriented segment of the population.

The lack of significant effects for effort expectancy and social influence across all countries reinforces this interpretation: early adopters tend to base their decisions on personal evaluations rather than external opinions and are rarely deterred by usability concerns. Furthermore, the fact that behavioral intention was unrelated to health status or health literacy underscores that current usage is not driven by acute medical need or advanced health literacy, but rather by interest, convenience, and technological exploration.

The cross-national consistency of these findings is particularly striking. Despite differences in health care systems, digital infrastructures, and culture, the same psychological and contextual factors influenced generative AI use in all countries surveyed. This suggests a shared adoption logic that transcends national boundaries—at least in the early stages of diffusion.

Looking ahead, these insights help illuminate how generative AI might transition from a niche tool to a widely used resource. As the technology becomes more embedded in everyday life, broader segments of the population—the so-called early and late majority—will likely demand stronger assurances of trustworthiness, safety, and added value. To enable responsible and inclusive adoption, it is therefore crucial to reduce digital access barriers, enhance transparency, and implement safeguards against health misinformation, especially for users with limited health literacy.

From a practical perspective, our findings suggest that communication strategies aiming to promote generative AI for health purposes should emphasize concrete benefits, ease of access, and even enjoyment. Rather than exclusively targeting individuals with chronic or urgent health needs, positioning generative AI as an engaging, low-barrier tool may broaden its appeal—reaching users who might otherwise be disengaged from traditional health information sources.

In sum, generative AI holds significant potential as a future health information resource—but its trajectory will depend on how well we understand and support the evolving needs of its users across different adoption phases and contexts.

## Supplementary material

10.2196/75648 Multimedia Appendix 1 Descriptive and reliability analysis of predictor variables.

10.2196/75648 Multimedia Appendix 2 Mean usage frequency of health information sources with 95% CI per country.

10.2196/75648 Checklist 1 CHERRIES checklist.

## Abbreviations

AI: artificial intelligence
CHERRIES: Checklist for Reporting Results of Internet E-Surveys
UTAUT2: Unified Theory of Acceptance and Use of Technology 2
WHO: World Health Organization
