# Supplementary material for: Predicting the Intention to Use Generative Artificial Intelligence for Health Information: Comparative Survey Study
Source: J Med Internet Res. 2026 Jan 28;28:e75648. doi: 10.2196/75648 (PMC12851524; doi:10.2196/75648)
Supplement: Multimedia Appendix 1 [file jmir-v28-e75648-s001.docx]

Supplement S1. Descriptive and reliability analysis of predictor variables.

| **Construct** | **Item** | **M** | **SD** | **Cronbachs**  **Alpha** |
| --- | --- | --- | --- | --- |
| AI usage intention  (7-point scale) | I intend to use generative AI for health information seeking. | 3.59 | 1.95 | .94 |
|  | I am open to trying generative AI when seeking health information online. | 4.06 | 1.99 |  |
|  | I will use generative AI for health information seeking frequently in the future. | 3.45 | 1.91 |  |
| Performance expectancy  (7-point scale) | I believe using generative AI for health-information seeking could be useful in my daily life. | 3.85 | 1.84 | .95 |
|  | I expect that using generative AI would help me find relevant information for my health. | 3.82 | 1.86 |  |
|  | I think using generative AI would save me time when researching health topics. | 3.98 | 1.91 |  |
|  | I perceive that using generative AI helps me take care of my health. | 3.51 | 1.84 |  |
| Effort expectancy  (7-point scale) | Learning how to use generative AI for health-information seeking seems easy for me. | 4.03 | 1.86 | .95 |
|  | Interacting with generative AI for health-information seeking appears clear and understandable. | 3.86 | 1.84 |  |
|  | I think using generative AI for health-information seeking is easy. | 3.87 | 1.85 |  |
|  | It seems easy for me to become proficient at using generative AI for health-information seeking. | 4 .00 | 1.88 |  |
| Social influence  (7-point scale) | People who are important to me think that I should use generative AI for health-information seeking. | 2.76 | 1.75 | .95 |
|  | People who influence my behavior think that I should use generative AI for health-information seeking. | 2.69 | 1.72 |  |
|  | People whose opinions I value prefer that I use generative AI for health-information seeking. | 2.74 | 1.75 |  |
| Hedonic motivation  (7-point scale) | Using generative AI for health-information seeking seems enjoyable to me. | 3.53 | 1.88 | .95 |
|  | I think using generative AI for health-information seeking could be fun. | 3.55 | 1.88 |  |
|  | Using generative AI for health-information seeking appears to be very entertaining. | 3.41 | 1.86 |  |
| Facilitating conditions  (7-point scale) | I have the resources necessary to use generative AI for health-information seeking (e.g., Computer, Tablet, Smartphone). | 5.04 | 2.04 | .84 |
|  | I have the knowledge necessary to use generative AI for health-information seeking. | 3.98 | 2.00 |  |
|  | I can get help from others when I have difficulties using generative AI for health-information seeking. | 3.88 | 1.99 |  |
|  | I have access to reliable internet connectivity, enabling me to use generative AI for health-information seeking efficiently. | 5.01 | 1.95 |  |
| Habit  (7-point scale) | The use of generative AI for health-information seeking has become a habit for me. | 2.55 | 1.79 | .95 |
|  | I regularly use generative AI for health-information seeking without consciously thinking about it. | 2.48 | 1.78 |  |
|  | I automatically turn to generative AI whenever I have questions about my health. | 2.48 | 1.82 |  |
| Health Literacy  (4-point scale) | I have good knowledge regarding health. | 2.80 | 0.82 | 0.90 |
|  | I am able to give ideas on how to improve health in my immediate surroundings (e.g., in family, leisure, or work context). | 2.78 | 0.84 |  |
|  | I can compare health-related information from different sources. | 2.95 | 0.81 |  |
|  | I can follow the advice given to me by healthcare personnel (e.g., nurse, doctor). | 3.12 | 0.76 |  |
|  | I can easily give examples of things that promote health. | 2.96 | 0.79 |  |
|  | I can judge how my actions affect the health of my immediate surroundings (e.g., in family, leisure, or work context). | 2.94 | 0.79 |  |
|  | I can find health-related information that is easy for me to understand. | 3.01 | 0.79 |  |
|  | I can judge how my behavior affects my health. | 3.13 | 0.75 |  |
|  | I can usually decide if health-related information is right or wrong. | 2.83 | 0.79 |  |
|  | I can provide reasons for the choices I make regarding my health. | 3.05 | 0.78 |  |
