# Supplementary figures and images for: Predicting the Intention to Use Generative Artificial Intelligence for Health Information: Comparative Survey Study
Source: J Med Internet Res. 2026 Jan 28;28:e75648. doi: 10.2196/75648 (PMC12851524; doi:10.2196/75648)

Supplement S2. Mean usage frequency of health information sources with 95 CI per country.
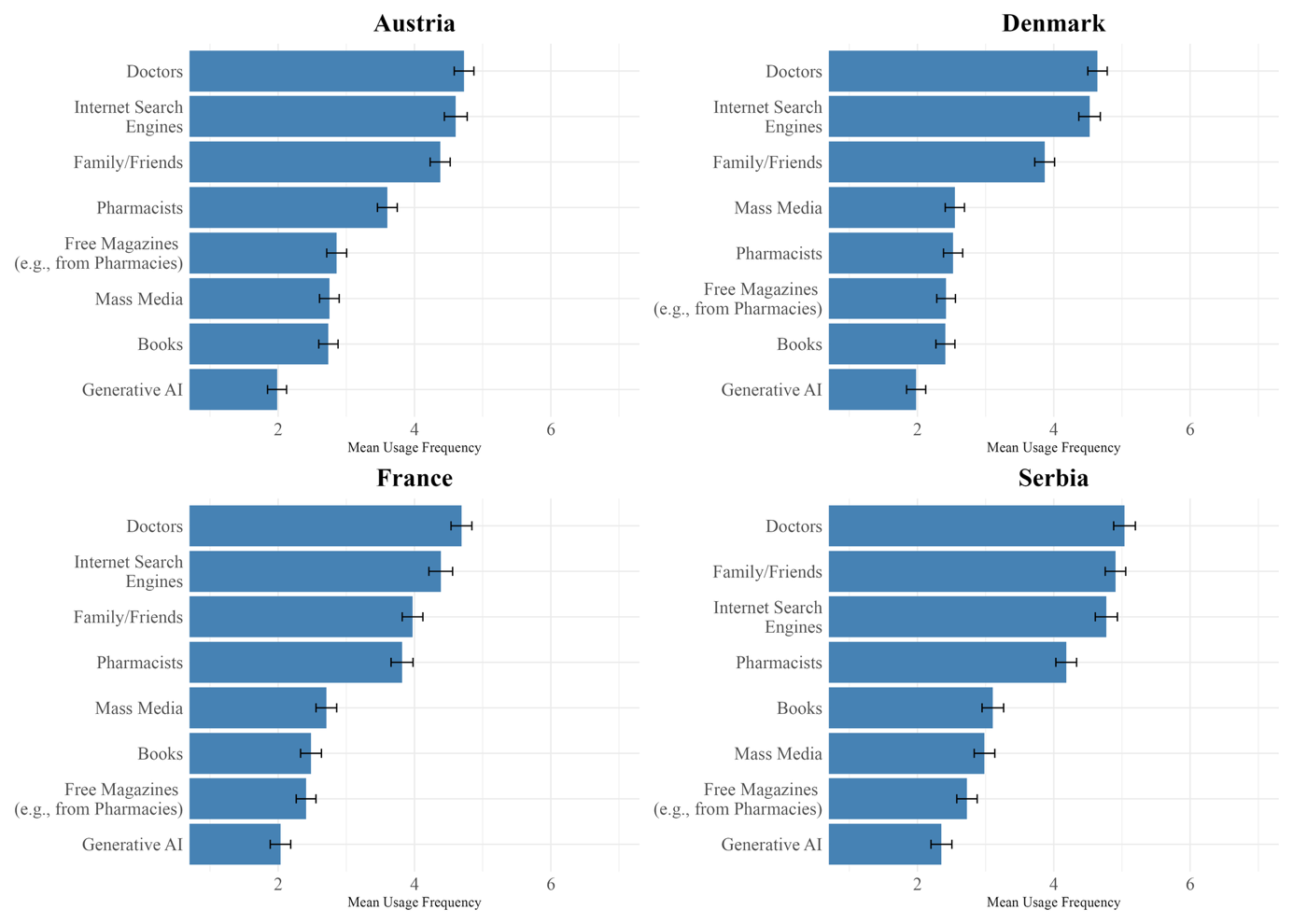

Supplement: Multimedia Appendix 2 [file jmir-v28-e75648-s002.docx]
