# Supplementary material for: Predicting the Intention to Use Generative Artificial Intelligence for Health Information: Comparative Survey Study
Source: J Med Internet Res. 2026 Jan 28;28:e75648. doi: 10.2196/75648 (PMC12851524; doi:10.2196/75648)
Supplement: Checklist 1 [file jmir-v28-e75648-s003.docx]

Supplement S3. Checklist for Reporting Results of Internet E-Surveys (CHERRIES).

| **Item Category** | **Checklist Item** | **Explanation** |
| --- | --- | --- |
| Design | Describe survey design | The study employed an online survey conducted in Austria, Denmark, France, and Serbia. Quotas were applied for age, educational attainment, and gender. Eligible participants were between 16 and 74 years of age. |
| IRB approval and informed consent process | IRB approval | Prior to data collection, the study received ethical approval from the Institutional Review Board of [blinded for review] (approval ID: [blinded]). |
|  | Informed consent | All participants provided informed consent electronically before taking part in the study. |
|  | Data protection | Data were collected in anonymized form, and no personal identifiers were recorded or stored. |
| Development and pre-testing | Development and testing | The survey was programmed in Qualtrics, using well-established and validated measurement instruments. The original questionnaire was developed in English and subsequently translated into German, French, Danish, and Serbian through a back-translation procedure to ensure accuracy and consistency across countries (translations are available on OSF). The usability and technical functionality of the questionnaire were tested in multiple rounds of pretesting. |
| Recruitment process and description of the sample having access to the questionnaire | Open survey vs. Closed survey | The study was conducted as an open survey; however, the survey link was distributed by the panel provider to participants preselected according to quotas for age, gender, and educational attainment. |
|  | Contact mode | Participants were invited to the study via email. |
|  | Advertising the survey | We collaborated with an online panel provider (Bilendi), who distributed the survey link to their participant pool. No additional advertising was conducted. |
| Survey administration | Web/E-Mail | The survey link was distributed via email and directed participants to the country-specific Qualtrics questionnaire, where data were automatically recorded. |
|  | Context | The panel provider’s participant pools in the respective countries were as follows:  Austria: n = 60,000  Denmark: n = 90,000  France = 815,000  Serbia = 15,100 |
|  | Mandatory/voluntary | Participation was voluntary. |
|  | Incentives | Upon completion of the survey, participants received a compensation of €1.50 from the online panel provider. |
|  | Time/date | Data were collected between September 9, 2024, and September 24, 2024. |
|  | Randomization of items or questions | Questions were presented in a fixed order, while items within questions were randomized. |
|  | Adaptive questioning | n.a. |
|  | Number of items | 102 items (survey part), 8 items (experimental part) |
|  | Number of pages | 33 pages (including intro, informed consent, and debriefing) |
|  | Completeness checks | Forced-response settings were applied to all questions. |
|  | Review step | Respondents were not able to review or change their answers, as no back button was provided. |
| Response rates | Unique site visitor | Each participant in Bilendi’s panel pool was assigned a unique identifier, which was recorded. |
|  | Participation rate (Ratio of unique visitors who agreed to participate/unique first survey page visitors) | Austria: 97.6%  Denmark: 97.3%  France: 98.2%  Serbia: 98.2% |
|  | Completion rate (Ratio of users who finished the survey/users who agreed to participate) | When including participants who were screened out due to quota being full or ineligibility, completion rates were:  Austria: 64.2%  Denmark: 52.0%  France: 21.7%  Serbia: 58.3%  When excluding screened-out participants, completion rates were:  Austria: 85.8%  Denmark: 85.9%  France: 84.3%  Serbia: 89.8% |
| Preventing multiple entries from the same individual | Cookies used | No cookies were used; instead, each participant’s unique panel ID ensured that multiple entries were not possible. |
|  | IP check | IP addresses were recorded but not used as unique identifiers. |
|  | Log file analysis | n.a. |
|  | Registration | n.a. |
| Analysis | Handling of incomplete questionnaires | Only fully completed questionnaires were included in the analysis, as incentives were provided by the panel provider exclusively to participants who finished the survey. |
|  | Questionnaires submitted with an atypical timestamp | Respondents who completed the survey in less than one-third of the median completion time (speeders) were excluded from the analysis. |
|  | Statistical correction | n.a. |
